# Disrupted iron regulation in the brain and periphery in cocaine addiction

**DOI:** 10.1038/tp.2016.271

**Published:** 2017-02-21

**Authors:** K D Ersche, J Acosta-Cabronero, P S Jones, H Ziauddeen, R P L van Swelm, C M M Laarakkers, R Raha-Chowdhury, G B Williams

**Affiliations:** 1 Department of Psychiatry, University of Cambridge, Cambridge, UK; 2 The Behavioural and Clinical Neuroscience Institute, The Wellcome Trust-MRC Institute of Metabolic Science, The John van Geest Centre for Brain Repair, University of Cambridge, Cambridge, UK; 3 German Center for Neurodegenerative Diseases (DZNE), Magdeburg, Germany; 4 Department of Clinical Neurosciences, University of Cambridge, Cambridge, UK; 5 CAMEO Early Intervention Service, Cambridgeshire and Peterborough NHS Foundation Trust, Cambridge, UK; 6 Radboud University Medical Center, Department of Laboratory Medicine, Nijmegen, The Netherlands; 7 Hepcidinanalysis.com, Nijmegen, The Netherlands

## Abstract

Stimulant drugs acutely increase dopamine neurotransmission in the brain, and chronic use leads to neuroadaptive changes in the mesolimbic dopamine system and morphological changes in basal ganglia structures. Little is known about the mechanisms underlying these changes but preclinical evidence suggests that iron, a coenzyme in dopamine synthesis and storage, may be a candidate mediator. Iron is present in high concentrations in the basal ganglia and stimulant drugs may interfere with iron homeostasis. We hypothesised that morphological brain changes in cocaine addiction relate to abnormal iron regulation in the brain and periphery. We determined iron concentration in the brain, using quantitative susceptibility mapping, and in the periphery, using iron markers in circulating blood, in 44 patients with cocaine addiction and 44 healthy controls. Cocaine-addicted individuals showed excess iron accumulation in the globus pallidus, which strongly correlated with duration of cocaine use, and mild iron deficiency in the periphery, which was associated with low iron levels in the red nucleus. Our findings show that iron dysregulation occurs in cocaine addiction and suggest that it arises consequent to chronic cocaine use. Putamen enlargement in these individuals was unrelated to iron concentrations, suggesting that these are co-occurring morphological changes that may respectively reflect predisposition to, and consequences of cocaine addiction. Understanding the mechanisms by which cocaine affects iron metabolism may reveal novel therapeutic targets, and determine the value of iron levels in the brain and periphery as biomarkers of vulnerability to, as well as progression and response to treatment of cocaine addiction.

## Introduction

Neuroscientific research in stimulant drug addiction has greatly advanced our understanding of the neurobiology of addiction. Although these advances have not yet translated into more effective treatments or prevention strategies, they have clearly demonstrated that addiction is a brain disorder.^1^ Critical to this has been accumulating evidence of the association of morphological brain changes with stimulant drug addiction, the most robust of these being the enlargement of the putamen, which is frequently seen in stimulant-addicted individuals.^2, 3, 4, 5, 6, 7, 8^ Preclinical animal models have shown that this abnormality is caused by drug effects, as the stimulant-induced decline in dopamine D2 receptors in the ventral striatum is directly linked with the volume increase in the dorsal striatum (putamen).^9^ This is thought to reflect a hypothesized ventral-to-dorsal progression in the behavioural shift from voluntary drug use to compulsive drug-taking^10^ and increased putamen volume may thus reflect a neural substrate of the transition to addiction. However, as it has also been observed in unaffected first-degree relatives of stimulant-addicted individuals^5^ and in patients with obsessive-compulsive disorder (OCD),^11^ putamen enlargement may partly represent a predisposing factor for compulsive behaviours.

Although these morphological brain changes in addiction have been well-characterized, the mechanisms by which stimulant drugs, whose primary pharmacological effect is to increase dopamine transmission, result in such changes remains unknown. One potential candidate mediator may be iron, which has a vital role in a many physiological processes, including in the synthesis of dopamine by providing energy for dopamine metabolism and storage.^12^ Given the pivotal role that iron plays in both health and disease, its metabolism is very tightly regulated. As an essential micronutrient, iron must be obtained from the diet and cannot be excreted (except by blood loss). Iron homeostasis is particularly critical in the brain because excess iron can result in neuronal death through the production of reactive oxygen species,^13^ and iron deficiency will impair dopamine synthesis and monoamine metabolism.^14^ Homeostasis is thus carefully controlled through various, highly complex transport systems and feedback loops.^15, 16^ Disruptions in the regulation of iron may therefore occur at various levels, resulting in a variety of different pathologies, prominent among which are neurodegenerative disorders.^17, 18^

Critical to the regulation of iron is the blood–brain barrier, which decouples iron levels in the periphery from the brain. Iron enters the brain as diferric transferrin (Tf) via the transferrin receptors (TfR1) and the divalent metal transporter 1 (DMT1).^19^ The transmembrane protein ferroportin 1 transports iron from the luminal to the abluminal side of a cell,^16, 20^ where it is stored as a ferritin, mainly in the oligodendrocytes but also in microglia and astrocytes.^21^ As the brain is one of the most metabolically active organs in the body, its demand for iron generally exceeds the rate of transferrin uptake, which means that the shortfall has to be covered from internal storage.^22^ To ensure that the internal storage is able to meet the demand, even in the event of inflammation or hypoxia, iron transport in brain parenchyma is regulated by the peptide hepcidin.^20, 23^ The regional distribution of iron in the brain is, however, not equal. Dopamine-rich brain regions like the basal ganglia are particularly susceptible to iron deposition, but the factors determining the regional distribution of iron remain elusive.^12^

Several lines of evidence suggest a dysregulation of iron homeostasis in stimulant drug addiction. First, regular use of stimulant drugs increases the permeability of the blood–brain barrier, allowing more iron to enter the brain parenchyma.^13, 24^ Second, in animal models stimulant exposure has been shown to be associated with iron deposition in the basal ganglia mainly in oligodendrocytes.^25^ Third, stimulant drugs impair innate immunity,^26^ rendering chronic stimulant users vulnerable to infection and chronic inflammation,^27^ disrupting peripheral iron homeostasis by reducing iron absorption or heam synthesis, and this is reflected by reduced serum iron and transferrin saturation.^28^ Finally, chronic stimulant drug use may change dietary preferences, particularly towards fatty foods,^29, 30, 31^ thereby affecting iron absorption due to lack of iron transporter and bioavailability.

We hypothesised that cocaine addiction is associated with disruptions in iron regulation, as reflected by increased iron concentration in the brain and reduced iron levels in the blood. We therefore sought to determine iron concentration in the brain, using quantitative susceptibility mapping,^32^ and in the periphery, using markers in circulating blood, in patients with cocaine addiction and age-matched healthy control volunteers. We predicted that increased levels of brain iron would be associated with the duration of cocaine use and basal ganglia volume in the cocaine-addicted patients.

## Materials and methods

### Study sample and procedures

We recruited 44 individuals (95% male) with a chronic history of cocaine use, meeting the DSM-IV-TR criteria for cocaine dependence, and 44 matched healthy control volunteers (93% male) without a history of drug or alcohol dependence. The diagnosis of cocaine dependence was ascertained using the Structured Clinical Interview for DSM-IV, and these individuals are subsequently referred to as cocaine use disorder (CUD). None of the control participants had ever met DSM-IV-TR criteria for substance dependence; for further details see Supplementary Material. All participants provided written informed consent before they underwent a medical review and psychiatric screening. Exclusion criteria included major medical or neurological illness, lifetime history of a psychotic disorder, history of a traumatic head injury, or any contra-indications to MR-scanning. Dietary iron intake was calculated from the Food Frequency Questionnaire (http://www.srl.cam.ac.uk/epic/nutmethod/FFQ.shtml). Diet-related variations in iron absorption were estimated using the algorithms developed by Hallberg and Hulthen.^33^ All participants provided blood samples for the analysis of iron proteins in serum (that is, ferritin, iron, transferrin), hepcidin-25, acute inflammation (that is, C-reactive protein (CRP)) and haematological status. This study was approved by the National Research Ethics Committee (12/EE/0519; PI: KDE).

### Neuroimaging data acquisition

All participants underwent magnetic resonance brain scans at the Wolfson Brain Imaging Centre, University of Cambridge (UK) using a 3T Siemens Magnetom Tim-Trio scanner. T1-weighted images (MPRAGE) and susceptibility-weighted images (SWI) were acquired for all participants. Brain scans were screened for normal radiological appearance by neuroradiologists. Scanning data from one control participant and three CUD patients were removed due to poor quality, leaving a total of 84 participants (43 controls, 41 CUD). Detailed neuroimaging methods are provided in the Supplementary Material.

### Statistical analysis

Data were analysed using a five-step strategy, as outlined below, and described fully in Supplementary Material. All statistical tests were two-tailed. In light of the multiple statistical tests, we applied a more stringent *P*-value, by dividing the initial *P*-threshold (0.05) by 10, resulting in a threshold of *P*<0.005. However, as this was an exploratory analysis, results reaching the *P*<0.05 threshold are also reported, though not referred to as significant in the discussion.

**Figure 1:**
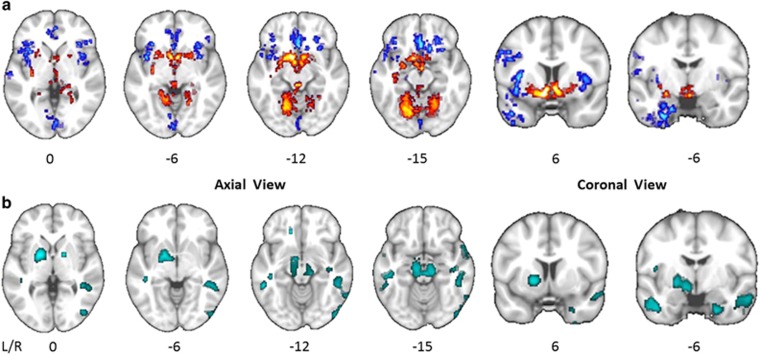
Whole brain maps of significant group differences. (**a**) Group comparison of modulated gray matter volume at whole brain level. Voxels colored in blue indicate brain areas in which patients with cocaine use disorder (CUD) have reduced gray matter volume compared with control volunteers, and voxels colored in red indicate brain areas in which CUD patients have abnormally increased gray matter volume. The statistical results are overlaid on the FSL MNI152 standard T1-image and the numbers beneath each section of the image refer to its position (mm) relative to the inter-commissural plane in standard stereotactic space. (**b**) Group comparison of iron concentration as estimated by quantitative susceptibility mapping (QSM) at whole brain level. Clusters colored in turquoise indicate greater iron concentration in CUD patients compared with control volunteers.

**Figure 2:**
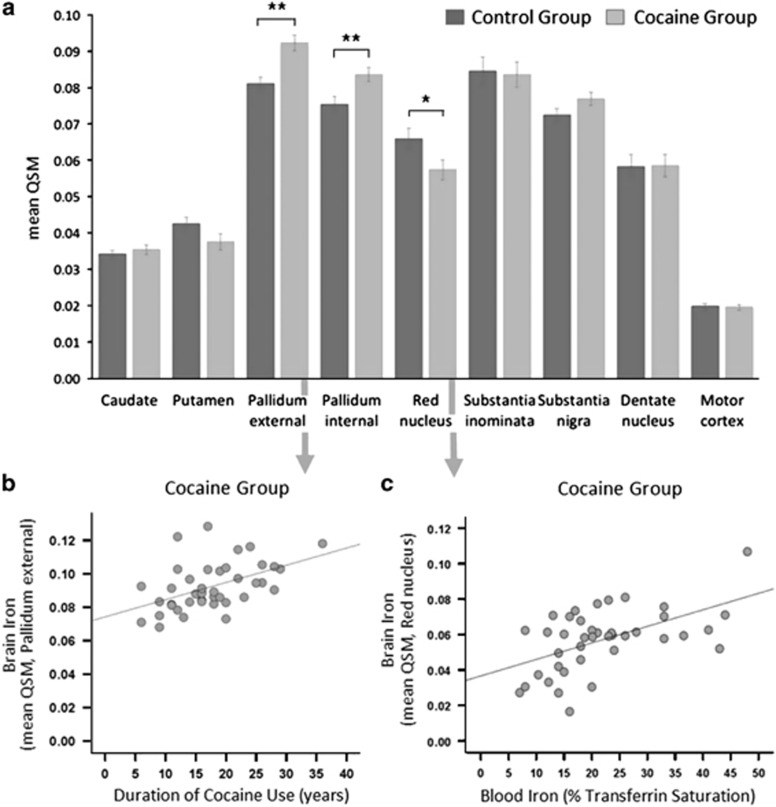
Regional group differences in iron concentration. (**a**) Group comparison of iron concentration in an region of interest (ROI)-based approach of iron-rich brain regions. CUD patients showed a significant increase in QSM of 14% in the globus pallidus external (GPe), of 11% in the globus pallidus internal (GPi), and a marginal increase of 6% in the substantia nigra. CUD patients further showed a significant reduction in QSM of 13% in the red nucleus; however, the 12% reduction in the putamen was non-significant (for statistical details see Supplementary Material). (**b**) Excessive iron accumulation in the GPe in CUD patients was significantly associated with the duration of cocaine use. (**c**) The reduction of iron concentration in the red nucleus in CUD patients was associated with a decrease in iron bound to transferrin in circulating blood. (Error bars denote s.e.m., **P < 0.005 and *P < 0.05). CUD, cocaine use disorder; QSM, quantitative susceptibility maps.

**Figure 3:**
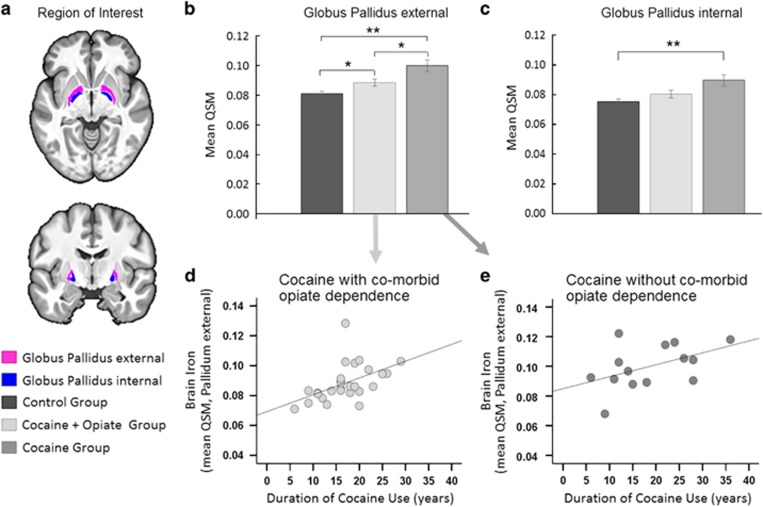
Cocaine-related abnormalities. (**a**) Illustration of the globus pallidus (GP), which was our region of interest. (**b**) *Post hoc* comparison of iron concentration in the GPe revealed significant differences between the groups, such that QSM levels in CUD patients with comorbid opiate dependence fell midway between the levels seen in healthy volunteers and CUD patients without comorbid opiate dependence. (**c**) Iron concentration in the GPi differed significantly between the groups, which was driven by significantly increased QSM in CUD patients without opiate dependence compared with healthy control volunteers. (Error bars denote s.e.m., **P < 0.005 and *P < 0.05). (**d**, **e**) In both CUD subgroups, excessive iron accumulation in the GPe was associated with the duration of cocaine use, supporting the notion that chronic cocaine use is implicated in the observed pathology. No relationships were observed between iron concentration in the GPi and the duration of cocaine use or with the duration of opiate use (for statistical details see text and Supplementary Material). CUD, cocaine use disorder; GPe, external GP; GPi, internal GP; QSM, quantitative susceptibility maps.

**Figure 4:**
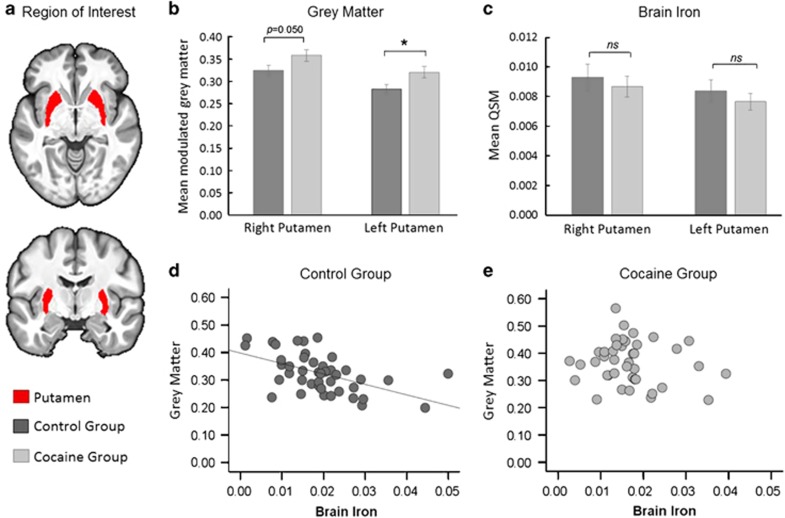
Possible pre-existing abnormalities. (**a**) Illustration of the putamen, which was our region of interest. (**b**) Group comparisons of gray matter volume showed a significantly increase in CUD patients compared with controls. (**c**) Levels of QSM were not measurable different between the groups. (Error bars denote s.e.m., NS indicates *P* > 0.05 and *P < 0.05). (**d**) Correlational analysis between gray matter volume (indexed by the mean of the modulated gray matter segment calculated over the FIRST ROI) and brain iron concentration (as indexed by QSM) are inversely correlated in control participants (*r*=−0.53, *P* < 0.001). (**d**) Gray matter volume was unrelated with QSM in CUD (*r*=−0.13, *P*=0.416). CUD, cocaine use disorder; QSM, quantitative susceptibility maps; ROI, regions-of-interest.

1. Demographics, clinical data and peripheral iron markers were examined in SPSS (v21) using independent-sample *t*-tests or Mann–Whitney *U*-tests. Chi-square or Fisher's exact tests were used for categorical data.
2. At whole brain level, gray matter volume comparisons were conducted on the MPRAGE images using FSL-VBM and CamBA for permutation testing. Quantitative susceptibility maps (QSM), a validated measure of brain iron concentration,^32^ were reconstructed from SWI data.^34^ Briefly, multi-channel complex data were combined using a modified adaptive algorithm.^35^ The combined phase images were unwrapped with a continuous Laplacian approach,^36^ and the local field was revealed through global extraction of the background field with spherical mean value filtering.^37^ QSM was estimated with the morphology-enabled, non-linear dipole inversion method,^38^ and the maps were warped into a study-wise space with a previously described processing stream^34^ using ANTs (v2.1). Finally, FSL Randomize (v2.9) was used for QSM statistical analysis (Figure 1).
3. Regions-of-interest (ROIs) were defined for nine iron-rich structures, which were manually traced on the QSM group template (Figure 2). In addition, to directly compare QSM in the putamen and globus pallidus (GP) against gray matter probabilities in the putamen, we applied an automated, reproducible algorithm for subcortical segmentation to the MPRAGE template using FSL-FIRST (Figures 3 and 4). Mean and median values for each ROI were imported into SPSS for group comparison and correlational analyses.
4. Correlational analyses were performed separately in each group to examine the relationships between iron concentration in both brain and periphery, with brain structure, age and duration of cocaine use.
5. Predictors of iron concentration in the GP were examined using a multiple regression model in SPSS with DSM-IV drug dependency status, smoking status, serum ferritin and transferrin saturation included as predictor variables.

## Results

### Demographics and peripheral iron markers

The two groups were matched in terms of age, gender, handedness, body mass, and alcohol consumption (Table 1). The groups did not differ on vital signs, indicating that CUD patients were not acutely intoxicated.

The estimated iron absorption calculated from dietary iron intake did not differ between CUD and controls (*t*_81_=−0.02, *P*=0.988). CUD patients had significantly lower serum iron levels than controls and significantly higher levels of the acute-phase reactants CRP and ferritin, indicative of inflammation (Table 1). Levels of transferrin (iron transporter) and hepcidin (iron regulatory protein) did not differ between the groups but CUD patients had significantly lower transferrin saturation, suggesting iron deficiency. Ferritin concentration was correlated with hepcidin levels in controls (*r*=0.40, *P*=0.008) and less strongly in CUD (*r*=0.38, *P*=0.016), suggesting that in patients the increase in ferritin may also be related to inflammation and other factors. The haematological profile in CUD patients further indicates mild iron deficiency with a microcytic blood picture (Table 1).

### Gray matter volume and brain iron concentration

Compared with the control group, gray matter volume at whole brain level was significantly increased in CUD in the putamen and the cerebellum, and significantly reduced in the insula, orbitofrontal, medial frontal, anterior cingulate and temporoparietal cortices (Figure 1a). Similarly, comparison of voxel-wise iron concentration (as indexed by QSM) at whole brain level, are shown in Figure 1b.

The ROI analysis showed significantly higher QSM concentration in CUD patients compared with controls in both segments of the GP (internal (GPi): *t*_82_=−2.88, *P*=0.005 and external (GPe): *t*_82_=−4.05, *P*<0.001). CUD patients also had reduced QSM in the red nucleus (*t*_82_=−2.14, *P*=0.036; Figure 2a), but this finding did not survive the more stringent threshold of *P*<0.005, which we applied due to multiple statistical testing (see also Supplementary Table S1).

To evaluate whether the increased putamen volume in CUD patients was associated with iron concentration in this region, correlations between gray matter volume and QSM were examined separately in both groups using (Figure 4, Supplementary Table S2). As putamen volume is expected to decrease with age (in contrast to QSM, which increases with age),^39^ age effects were also examined. In controls, putamen gray matter volume was significantly negatively correlated with QSM (*r*=−0.53, *P*<0.001) and age (*r*=−0.37, *P*=0.015) but these relationships were not seen in CUD (QSM: *r*=−0.13, *P*=0.416; age: *r*=0.05, *P*=0.770). In both groups, putamen volume was also unrelated to QSM in the GP, and was not associated with the duration of cocaine use (*r*=0.19, *P*=0.229).

There were no group differences with respect to GP or caudate volume, and no significant volume — QSM relationships in the GP (Supplementary Material).

### Brain iron accumulation as a function of age and cocaine use

Consistent with prior work in healthy ageing,^34, 40^ in controls iron concentration was correlated with age in the putamen (*r*=0.67, *P*<0.001), red nucleus (*r*=0.64, *P*<0.001), substantia inominata (*r*=0.49, *P*=0.001), substantia nigra (*r*=0.48, *P=*0.001), motor cortex (*r*=0.43, *P*=0.005), and caudate nucleus (*r*=0.39, *P*=0.010). However, in CUD patients, these correlations were only seen in the motor cortex (*r*=0.44, *P*=0.005) and substantia nigra (*r*=0.43, *P*=0.005), suggesting that the pattern of iron accumulation with normal ageing is altered in cocaine addiction.

As shown in Figure 2b, the duration of cocaine use correlated strongly with QSM in the GPe (*r*=0.49, *P*=0.001), the substantia nigra (*r*=0.34, *P*=0.028) and dentate nucleus (*r*=0.33, *P*=0.034), but not in the GPi (*r*=0.28, *P*=0.069). QSM in the red nucleus was significantly correlated with low transferrin saturation in CUD patients (*r*=0.54, *P*<0.001, Figure 2c) but not in controls (*r*=-0.08, *P*=0.628). Only in the dentate nucleus was QSM correlated with transferrin saturation in both groups (CUD: *r*=0.43, *P*=0.005, controls: *r*=0.31, *P*=0.045). For further details, see Supplementary Material.

### Predictors of iron accumulation in the GP

Multiple regression revealed that a third of the variance (34%) of QSM in the GPe was explained by drug dependency (*R*^2^ =0.34; F_7,74_=5.1832, *P*<0.001), with cocaine dependence (*β*=0.57, *P*=0.036) and the absence of opiate dependence (*β*=−0.47, *P*=0.001) being the strongest predictors in the model. For the GPi, the same model explained only 18% of the variance of QSM (*R*^2^=0.18; *F*_7,74_=2.27, *P*=0.038) with the absence of opiate dependence being the only significant predictor (*β*=−0.37, *P*=0.018); see Supplementary Material for details.

Given the strong negative relationship with opiate dependence, the CUD group was divided *post hoc* into two subgroups with and without comorbid opiate dependence (CUD+O, *n*=27; CUD−O, *n*=14). There were no significant demographic differences between the CUD-subgroups. As shown in Figure 3b, one-way ANOVA revealed significant group differences in QSM in GPe (F_2,81_=13.21, *P*<0.001) such that QSM was increased in both CUD-subgroups compared with controls (both Tukey's *P*<0.05) and significantly increased in CUD−O compared with CUD+O (*P*=0.013). As shown in Figures 3d and e, the duration of cocaine use was significantly correlated with QSM in CUD+O (*r*=0.48, *P*=0.017) and in CUD−O (*r*=0.54, *P*=0.047) (Fisher's *z*-score=−0.34, *P*=0.737). The group effect in QSM in the GPi (*F*_2,81_=6.87, *P*=0.002) was driven by significantly higher levels of QSM in CUD−O compared with controls (*P*=0.001). Differences between CUD+O and controls (*P*=0.253) and CUD+O and CUD−O (*P*=0.070) were non-significant (Figure 3c). No relationships were found between QSM in GPi and the duration of cocaine use or the duration of opiate use (Supplementary Material).

## Discussion

We demonstrate for, we believe, the first time in humans that chronic cocaine use is associated with excessive iron accumulation in the brain, which is localised to the GP and correlates strongly with the duration of regular cocaine use, but is unrelated to GP volume. We further report a significant reduction in serum levels of transferrin-bound iron (transferrin saturation), which was associated with low iron concentration in the red nucleus. These findings suggest that iron regulation is disrupted in cocaine addiction. Although we replicated the established findings of putamen enlargement in CUD, we did not find supporting evidence for this being related to iron accumulation either in the GP or the putamen, suggesting that both are separate, co-occurring morphological changes in cocaine addiction (Supplementary Table S3).

### Iron accumulation in the GP: a putative consequence of cocaine exposure?

Excessive brain iron accumulation is a recognised pathological change in neurodegenerative diseases and conditions caused by inherited abnormalities of iron metabolism, but not one that has been described previously in cocaine addiction. Indeed, the highly localized iron accumulation in the GP in CUD patients is reminiscent of the ‘eye of the tiger' sign on T2-weighted magnetic resonance imaging in pantothenate kinase-associated neurodegeneration (formerly Hallervorden-Spatz syndrome); a rare, rapidly progressive neurodegenerative disorder with childhood onset and prominent extrapyramidal symptoms. This condition is characterised by disturbances of systemic iron metabolism caused by mutations in the *PANK2* gene.^41^ It thus demonstrates a clear link between iron dysregulation, basal ganglia dysfunction and excessive iron accumulation selectively in the GP, which may be very relevant to understanding our finding in CUD, though the precise mechanisms of how this arises and relates to the progression of the clinical syndrome remain to be determined.

High concentrations of iron are generally seen in the oligodendrocytes in dopamine-rich basal ganglia structures, the highest being in the GABAergic interneurons of the GP. Although the GP itself is not a dopamine-rich structure, its function within the basal ganglia network is, however, dependent on dopaminergic inputs.^42, 43, 44^ Unlike other regions such as the putamen, which accumulate iron slowly but steadily throughout the lifespan, iron concentration in the GP increases rapidly during the first two decades of life (when iron is essential for growth and myelination), and then begins to plateau around the age of 30 years.^21^ GP iron concentration in our CUD group not only exceeded that of age-matched healthy volunteers (mean age 40 years), but also correlated strongly with the duration of cocaine use (Figure 2). Preclinical work in monkeys has shown a similarly selective increase in iron concentration in the GP following exposure to methamphetamine, at a magnitude 2.5 times that in control animals.^25^ In humans, bilateral GP infarction is a common symptom of cocaine overdose.^45, 46, 47^ Taken together, these findings suggest that this excess iron accumulation in the GP is a regionally specific effect of stimulant drug exposure.

### Mechanisms and functional implications of excess iron accumulation in the GP

Evidence from animal models and human studies revealed that the stimulant-induced iron increase in the GP occurs with a delay of up to 18 months after exposure.^25^ Although iron accumulation has primary been regarded as a dopamine-driven mechanism,^48^ this abnormal iron increase in the GP appears to reflect compensatory GABAergic adaptations to excessive stimulant-induced dopaminergic excitation in the striatum.^49, 50^ However, the putative involvement of GABA and glutamate in iron homeostasis^50^ might also play a role in this process. Though iron concentrations in CUD patients were significantly increased in both segments of the GP, they were only related to the duration of cocaine use in the GPe. This cocaine-related iron accumulation further appeared to be attenuated by comorbid opiate addiction (Figure 3); a finding that requires further investigation. Consistent with previous work,^51^ the majority of our CUD+O reported using heroin in particular to alleviate cocaine-induced over-excitability and to enhance their control over their cocaine use. Whether these experiences are related to the attenuated iron increase in the GP remains to be determined.

The GPe and the subthalamic nucleus form the so-called indirect striatal pathway,^52^ which has been hypothesized to act as ‘brake' for inhibiting ongoing behaviour^53^ and to mediate learning of aversive outcomes^54, 55^—functions that are substantially impaired in stimulant-dependent individuals.^56, 57^ Owing to its ‘strategic location', the GPe has been suggested to exert a powerful inhibitory control on basal ganglia output structures,^58^ which are implicated in habit learning and automaticity.^59^ The indirect striatocortical pathway is thought to be modulated via striatal dopamine receptor type 2 function, which has been shown to decline following repeated stimulant drug exposure^9, 60^ and to underlie to compulsive drug-taking in addicted individuals.^61, 62^ Intriguingly, selective damage of the GP has repeatedly been associated with compulsive behaviours unrelated to drug taking,^63, 64, 65^ it is thus conceivable that GP dysfunction contributes to disruptions in the balance between goal-directed and habitual action control that characterises cocaine addiction.^10^ However, despite existing histological evidence of the stimulant-induced increase in the GP^25^ and preliminary preclinical evidence suggesting that an imbalance between direct and indirect striatal pathway activity might mediate the transition to addiction,^66^ the underlying mechanisms and functional effects of iron dyregulation on addictive behaviour require further investigation.

### Iron dysregulation in the periphery in cocaine addiction

We found evidence of dysregulation of peripheral iron metabolism in our CUD sample with significantly reduced levels of iron and transferrin saturation (Table 1). Given that transferrin is the major iron transporter protein, one would anticipate increased rather than decreased transferrin saturation in mild iron deficiency. However, during acute inflammation (as reflected by increased CRP levels, and abnormal eosinophil, lymphocyte and neutrophil counts), ferritin levels may increase disproportionately due to hepatic ferritin production, while iron release remains proportionate to the cellular ferritin content, resulting in low transferrin saturation as seen in the present study. The rather modest increase in ferritin during inflammation in CUD patients further supports the notion of peripheral iron dysregulation.

As iron crosses the blood–brain barrier bound to transferrin,^67^ the correlation between peripheral transferrin saturation and low iron levels in the red nucleus in CUD patients also concurs with the notion of peripheral iron deficiency (Figure 2c). In conditions of iron overload, the exact opposite pattern has been reported, namely excessive iron accumulation in the red nucleus and increased transferrin saturation in the periphery.^68^

### A possible mediating role of peripheral inflammation in central iron accumulation?

Given that we see elevated serum CRP and ferritin levels in the CUD, it may be that inflammation is a mediator of the increased iron brain iron concentration. Although we found no significant correlations between CRP levels and QSM, this does not rule out this possibility, as QSM cannot separately detect the ferritin l-isoform, which is associated with inflammation. Ferritin has two dominant isoforms, light polypeptide (l-ferritin) and heavy polypeptide (H-ferritin). H-ferritin has a critical regulatory role in iron metabolism, whereas the physically more stable l-ferritin is more involved in iron storage of iron.^69^ H-ferritin is involved myelination and adenosine triphosphate (ATP)^70^ and predominates in the young adult brain. However with aging, l-ferritin levels in the brain increase with the accumulation of myelin debris and the breakdown of neuromelalin,^13^ and l-ferritin is thought to play a critical role in neurodegeneration.^69^ To examine the association between iron accumulation in the brain and inflammation, it is necessary to specifically measure l-ferritin, which is not possible at present with QSM, and this remains an important area of future research.

### Putamen enlargement is unrelated to iron levels

Though we replicated the finding of putamen enlargement in chronic stimulant drug users^2, 3, 4, 5, 6, 7, 8^ in our CUD group, we did not find supporting evidence for relationships between volumetric changes and abnormal iron concentration. Despite the group differences in putamen volume, iron concentration in the putamen did not significantly differ between the two groups (Figure 4). However, unlike the control group, CUD patients showed no correlation between putamen iron and age. Clearly, the pattern seen in CUD does not seem to accord with either a normal ageing^39^ or a neurodegenerative picture.^71^ Given that enlargement of the putamen has also been observed in their unaffected biological siblings,^5^ it likely represents a vulnerability factor to, rather than a consequence of, cocaine addiction.

### Strengths and weaknesses

The strengths of the study are its methodology, the simultaneous examination of central and peripheral iron and the relatively large sample size. For the measurements of brain iron QSM has proven superior to R2,^40^ and has been cross-validated against both other MR techniques and post-mortem iron measurements.^32^ Although QSM primarily indexes iron concentration, it might also capture other processes such as lower myelin levels,^72, 73^ it remains unclear to what extent such alterations might have a role in CUD and in the present results. Limitations include the cross-sectional design, the lack of behavioural assessments and the absence of measures iron absorption/transport, such as DMT1 and ferroportin (particularly iron absorption through gut mucosa), which would help determine the specific disruptions in iron regulation in CUD patients.

### Implications and outlook

Over the last three decades there has been a major conceptual shift in our understanding of drug addiction, from simply being a behavioural problem to a brain disorder.^1, 74^ There have also been significant advances in addressing the basic question of causality, namely as to whether the observed brain changes in addicted individuals were causal or consequential to drug addiction. However, we know surprisingly little about the neurobiological mechanisms by which stimulant drugs interact with vulnerability factors leading to the observed neuropathology. In this study, we provide first evidence of a potential mechanism, which seems to arise consequent to cocaine exposure, namely the dysregulation of iron metabolism. Future research is warranted to identify the precise mechanisms by which stimulant drugs interact with iron regulation, that is, changing the permeability of the blood-brain-barrier, altering intracellular iron trafficking, or disrupting cellular iron management. Another possibility that requires further exploration is the role of peripheral inflammation in CUD, and its contributory or mediating role in iron accumulation in the brain, given the growing evidence of inflammatory conditions affecting iron homeostasis through a process of translational regulation of ferritin.^75^

The demonstration of iron dysregulation in cocaine addiction raises several important questions including, how this develops over time, whether iron deficiency increases the vulnerability to developing stimulant addiction, contributes to the persistence of the disorder, or leads to the long-term sequelae such as accelerated brain ageing and behavioural and motor rigidity that continue in recovery. These will require longitudinal studies to evaluate the temporal course and stability of imbalances in iron regulation, and relate them to other key aspects of the illness. A more critical question that follows from the above is whether impaired iron regulation might be a biomarker of disease progression or a therapeutic target for cocaine addiction, either through addressing peripheral iron deficiency to modify disease course, or by slowing or reversing the central accumulation of iron. Given that stimulant addiction remains a significant public health problem associated with considerable harm and morbidity, with few pharmacological treatments and only modestly successful psychosocial treatments, elucidating this mechanism may offer the possibility of developing some much needed new interventions.

## Figures and Tables

**Table 1:** Demographics, haematological and peripheral iron measures

*Demographics*	*Control Group*	*Cocaine Group*	*Group Comparison*		
	*Mean (±s.d.)*	*Mean (±s.d.)*	t *or* U	P-*value*	
Gender ratio (male: female)	40: 3	39: 2	Fisher's	1.000	
Handedness (right: left: ambidextrous)	37: 5: 1	34: 6: 1	Fisher's	0.875	
Age (years)	41.7 (±10.6)	40.5 (±7.6)	0.60	0.553	
Body mass index	24.8 (±3.1)	23.4 (±4.1)	1.75	0.084	
Alcohol consumption (AUDIT score)	3.9 (±1.9)	4.0 (±4.8)	1.24	0.222	
					
*Selected markers related to iron status*					
Dietary iron (mg)	11.6 (±3.7)	13.4 (±6.2)	−1.66	0.100	
Serum iron (μmol l^−1^)	17.9 (±5.9)	12.8 (±5.5)	4.03	<0.001	
Serum ferritin (μg l^−1^)	65.0 (±46.1)	103.2 (±75.2)	−2.80	0.008	
Serum transferrin (g l^−1^)	2.8 (±0.4)	2.6 (±0.4)	1.13	0.332	
Transferrin saturation (%)	29.1 (±9.6)	21.9 (±10.3)	3.32	0.001	
Serum hepcidin-25 (nm)	3.4 (±3.8)	3.7 (±3.0)	710.5	0.125	
					
*Selected inflammatory marker*					
C-reactive protein (mg l^−1^)	2.5 (±2.6)	9.2 (±9.8)	436.5	<0.001	
					
*Selected haematology markers*					
Haemoglobin (g l^−1^)	143.9 (±7.5)	136.9 (±12.1)	3.15	0.002	
Haematocrit (l l^−1^)	0.4 (±0.02)	0.4 (±0.04)	2.43	0.018	
Red blood cells (10^12^ per l)	4.8 (±0.3)	4.6 (±0.4)	−3.37	0.001	
White blood cells (10^9^ per l)	5.9 (±1.3)	7.4 (±2.6)	1.73	0.088	
Eosinophils (%)	1.8 (±1.1)	3.3 (±1.7)	−4.73	<0.001	
Lymphocytes (%)	28.3 (±6.4)	33.7 (±10.2)	−2.92	0.005	
Monocytes (%)	7.50 (±1.8)	8.6 (±2.5)	−2.31	0.024	
Neutrophils (%)	61.8 (±7.0)	53.7 (±11.7)	3.82	<0.001	
Basophils (%)	0.6 (±0.3)	0.7 (±0.4)	−0.90	0.369	
Mean cell volume (Fl)	89.4 (±4.6)	88.8 (±6.4)	0.52	0.602	
Mean cell haemoglobin (Pg)	30.3 (±1.7)	29.7 (±2.4)	1.18	0.242	
Mean corpuscular haemoglobin concentration	338.5 (±6.9)	334.63 (±8.3)	2.27	0.026	
					
*Other biomarkers*					
Creatinine (μmol l^−1^)	79.9 (±14.4)	79.7 (±16.1)	0.04	0.970	
Total bilirubin (μmol l^−1^)	14.2 (±9.8)	6.5 (±3.3)	302.5	<0.001	
Conjugated bilirubin (μmol l^−1^)	3.9 (±2.5)	2.5 (±1.3)	555.5	0.003	

Abbreviations: Std., standard deviation.

Means and standard deviations appear in parentheses.
