# Supplementary material for: Disrupted iron regulation in the brain and periphery in cocaine addiction
Source: Transl Psychiatry. 2017 Feb 21;7(2):e1040–. doi: 10.1038/tp.2016.271 (PMC5438021; doi:10.1038/tp.2016.271)
Supplement: Supplementary Information [file tp2016271x1.doc]

**Disrupted iron regulation in the brain and periphery in cocaine addiction**

Supplementary Material

Karen D Ersche, Julio Acosta-Cabronero, P Simon Jones, Hisham Ziauddeen, Rachel van Swelm, Coby M.M. Laarakkers, Ruma Raha-Chowdhury, Guy B Williams

🖂 Correspondence: [ke220@cam.ac.uk](mailto:ke220@cam.ac.uk)

**SUPPLEMENTARY METHODS**

**Study sample**

All volunteers consented in writing before undergoing a medical review and a psychiatric screening procedure using the Mini International Neuropsychiatric Inventory (MINI)^1^. Psychopathology in drug users was further evaluated using the Structured Clinical Interview for DSM-IV (SCID)^2^. A medical or neurological illness, autoimmune or metabolic disorder, lifetime history of a psychotic disorder, history of a traumatic head injury, HIV-infection, or any contra-indications to MR-scanning led to exclusion from the study. All participants completed the National Adult Reading Test (NART)^3^ to provide an estimate of verbal IQ, and the Alcohol Use Disorders Identification Test (AUDIT)^4^ to assess alcohol consumption, drinking behaviours, and alcohol-related problems. Control participants were also asked to complete the Drug Use Questionnaire (DAST-20)^5^ to provide a brief indicator of drug use and related problems. Urine samples were tested for undeclared drugs and breath tests were also used to screen for acute alcohol intoxication. All samples provided by patients with cocaine use disorder (CUD) tested positive for cocaine, and urine samples provided by healthy volunteers were all negative. The study was approved by the National Research Ethics Committee (10/H0306/69; PI: KDE).

On average, CUD patients reported using cocaine/crack-cocaine for 17 years [±6.8 standard deviation (SD)]. Of the total CUD sample, 29 also met DSM-IV-TR criteria for opiate dependence, 16 for cannabis dependence and three for alcohol dependence. Ninety-six percent reported smoking tobacco, but DSM-IV-TR nicotine dependence was not assessed. In terms of medication 13 CUD patients were prescribed methadone (mean dose 51.9ml, SD±15.5), 10 were prescribed buprenorphine (mean dose 7.0 ml, SD±4.8), two were prescribed benzodiazepines and one was prescribed steroids for the treatment of asthma. CUD participants were recruited from local drug treatment services and by word of mouth. Healthy volunteers were recruited by advertisement from the local community and the Cambridge BioResource volunteer panel. None of the control participants had ever met DSM-IV-TR criteria for substance dependence. Five percent reported smoking tobacco on a regular basis. Overall, drug-taking and drinking was low in the control group, as reflected by low scores on both the DAST-20 (mean score 0.38, SD±0.6) and the AUDIT (mean score: 3.9, SD±1.9). None of the healthy control participants were on any regular prescribed medication.

**Dietary and peripheral biomarkers**

Dietary iron intake was calculated from the Food Frequency Questionnaire (FFQ; (http://www.srl.cam.ac.uk/epic/nutmethod/FFQ.shtml), which all participants completed prior to scanning. The FFQ is a validated, widely-used tool that determines individuals’ habitual dietary food intake by measuring the frequency with which food items have been consumed over the past year^6^. FETA software was used to calculate nutrient intake from the FFQ responses (www.srl.cam.ac.uk/epic/epicffq/). Variations in iron absorption caused by the intake of nutrients that enhance (e.g. ascorbic acid) or inhibit (e.g. tanic acid) iron uptake from food, were estimated using the algorithms developed by Hallberg and Hulthen^7^.

All participants provided non-fasting venous blood samples. One sample was immediately analysed at the Department of Pathology at Cambridge University Hospitals (UK) for iron proteins in serum (i.e. ferritin, iron, transferrin), acute inflammation [i.e. C-reactive protein (CRP)] and haematological status. A second sample was stored as serum at -80°C and sent to the Department of Laboratory Medicine at Radboud University Medical Center at Nijmegen (Netherlands) for the analysis of serum hepcidin-25, a key regulator of iron metabolism [performed using a combination of weak cation exchange chromatography and time-of-flight mass spectrometry^8^].

**Neuroimaging data acquisition**

All participants underwent magnetic resonance (MR) brain scans at the Wolfson Brain Imaging Centre, University of Cambridge, UK using a 3T Siemens Magnetom Tim Trio scanner with a Siemens 32-channel phased-array head coil (Siemens Healthcare, Erlangen, Germany). T1-weighted MR scans were acquired using a magnetization-prepared rapid acquisition gradient-echo (MPRAGE) sequence with the following imaging parameters: repetition time (TR) = 2300 ms, echo time (TE) = 2.98 ms, inversion time = 900 ms, flip angle = 9°, FOV = 240 x 256 x 176 mm^3^ with 1 x 1 x 1 mm^3^ voxel size). Susceptibility weighting (SW) consisted of complex MRI signals from a 3D fully-flow compensated gradient-recalled echo pulse sequence with TR = 29 ms, TE = 20 ms, flip angle = 17˚, FOV= 256 x 240 mm^2^ with 1 x 1 mm^2^ in-plane resolution, and 72 straight axial slices with 2-mm thickness for whole-brain coverage; bandwidth was set to 80 Hz per pixel. GRAPPA was enabled with acceleration factor of 2 and 24 reference lines. All images were screened for normal radiological appearance by a specialist in neuroradiology. One MPRAGE scan of a CUD patient was removed due to excessive movement; and on similar grounds, SWI data from three CUD patients and one control participant were also excluded, leaving 84 participants (43 controls and 41 CUD) in the final sample.

**Neuroimaging data analyses**

Although the hypotheses of the present study are region-specific (i.e. basal ganglia), group comparisons were first performed at the whole brain level to reproduce previously published results in drug addiction and ageing with independent data^9-11^. Quantitative susceptibility mapping was our method of choice because, as a phase-based method, it is more sensitive for the detection of brain iron accumulation than the R2 method^12^, which has previously been reported in the literature^13^.

We performed two regional approaches: first, we examined group differences in iron-rich structures; second, we compared volumetric and QSM estimates from putamen and pallidum.

*Pre-processing of grey matter volume*

In order to qualitatively compare QSM with structural results, grey matter probability maps from 84 participants (43 controls and 41 CUD) were processed using FSLVBM (<http://www.fmrib.ox.ac.uk/fsl/fslvbm>, Version 5.0) and SPM12 (http://www.fil.ion.ucl.ac.uk/spm). Brain masks were created following tissue segmentation using SPM12 segment function. Brain was identified as regions of segments c1, c2 and c3 greater than 0.5 but excluding regions of c4, c5 and c6 greater than 0.15. Subsequently, the standard FSLVBM pipeline was followed with brain masked T1 images segmented using FSL FAST^14^ as part of the fslvbm_2_template step. The resulting grey matter partial volume images were aligned to MNI-152 standard space using the affine registration tool FLIRT, followed by nonlinear registration using FNIRT, which uses a b-spline representation of the registration warp field. A study-wise grey matter template was computed, to which native grey matter images were then nonlinearly re-registered. To correct for local expansion or contraction, the co-registered partial-volume images were modulated by multiplication with the Jacobian of the warp field. For subsequent voxel-wise analyses, the modulated segmented images were then smoothed with an isotropic Gaussian kernel with full width at half maximum (FWHM) = 2.3 mm.

*Quantitative susceptibility mapping (QSM) reconstruction*

Complex SWI data from each receiver channel were combined offline with an adaptive method^15^ and were post-processed [full details are described elsewhere^11^]. Briefly, a brain mask was created from the combined magnitude image using FSL BET2^14^ with fractional threshold 0.1. The combined phase maps were unwrapped with a Laplacian method^16^, and the local field was inferred from the global extraction of background contributions with the spherical mean value filtering approach^17^ through convolution with a 5-mm radius sphere. QSM was estimated using the iterative, morphology-enabled, nonlinear dipole inversion (MEDIN) method with dynamic model error reduction (MERIT)^18^ − source code available from <http://weill.cornell.edu/mri/pages/qsm.html> − to balance extended data smoothness and local fit with 500 internal iterations and normalized residual tolerance of 0.01. A Lagrangian multiplier of 1,000 was optimized using a previously described empirical approximation^11^. Due to QSM’s polarity, to reduce the impact of spurious contamination at the grey matter–white matter interface, median values were used for regional analysis.

As in our previous study, QSM was normalized to a reference region exhibiting low global relative variance, and was compared with non-normalized data^11^. Age-related effects were identified in the control group using both methods but did not differ significantly. In addition, using the complete cohort, we did not find any significant differences between non-normalized and normalized data, suggesting the QSM effect sizes in drug addiction are also much greater than the magnitude of the reference adjustment. In order to facilitate comparability with future studies, the present results were thus reported with non-normalized data.

As in our previous ageing study^11^, all bulk susceptibility maps were warped into an average space using an ANTs template generation routine^19^ [version 2.1.0 (http://stnava.github.io/ANTs)]. First, MPRAGE images were N4-ITK^20^ radio-frequency bias-corrected and were then warped iteratively with the diffeomorphic Greedy-SyN transformation model to build a study-wise anatomical template. The pipeline consisted of four rigid-then-affine iterations followed by six full runs of a nonlinear routine to ensure stable convergence. Each N4 bias-corrected susceptibility-weighted magnitude image was then affine matched to its corresponding MPRAGE. These transformations were concatenated to align all QSMs with the study-wise anatomical template. Subsequently, a QSM group-average template was calculated from all spatially normalized QSMs. The QSMs reported here were not further adjusted to preserve original values.

In order to robustly test for non-haeme, parenchymal QSM differences, signals from ventricles and major vessels were removed. This involved skull stripping the MPRAGE template using the SPM segment function as described previously, applying this mask to the QSM template and manually editing the QSM template to remove any remaining non-brain voxels. The resulting template was binarised to create a QSM template mask, which was then applied to the individual QSMs. Finally, the resulting maps were thresholded at 0.0012 (the group mean 10^th^ percentile positive value) to minimise the influence of diamagnetic tissue and were smoothed by a 3-mm standard deviation Gaussian kernel.

*Voxel-wise Analyses*

The smoothed, positively thresholded QSM maps from participants were used in the whole-brain group comparisons. Statistical differences were compared using a GLM equivalent to a two-sample t-test design, and also an ANCOVA GLM including age as a covariate. The significances of the results were corrected using permutation testing in FSL Randomize with threshold-free cluster enhancement correction and 5000 permutations. Results were deemed significant with corrected p-values less than 0.05.

*Region-based Analyses*

ROIs were manually traced on the masked QSM group template. The QSM template was positively thresholded at 0.03 which was a level that partially isolated the high phase QSM kernels of the regions of interest except for the motor cortex to which a lower threshold of 0.008 was applied. Each cluster in the thresholded image was labelled using the FSL cluster function and those clusters representing the regions of interest were extracted as shown in supplementary Figure S1. Manual edits were made to separate clusters in the substantia nigra from the red nucleus. The pallidum cluster was further thresholded at 0.06 at which level internal and external regions could be separated.

Additionally, anatomical subcortical ROIs were created by processing the MPRAGE group template through FSL FIRST. In order to minimize edge effects, all anatomical FIRST ROIs were eroded by 1mm in 3D.

**Multiple regression model**

Multiple regression models were performed to investigate whether QSM in the GP can be predicted by drug dependency (assessed by DSM-IV-TR criteria), tobacco smoking status (measured by self-report), peripheral iron biomarkers such as levels of serum ferritin (measured in the blood) and transferrin saturation (calculated from serum iron and transferrin). Dependency and tobacco smoking status were dichotomous variables (D1: not cocaine-dependent, cocaine-dependent; D2: not opiate-dependent, opiate-dependent; D3: not cannabis-dependent, cannabis dependent; D4: not alcohol-dependent, alcohol-dependent; D5: smoker, non-smoker); ferritin and transferrin saturation were continuous variables. All predictor variables were entered at the same time in the model.

**Corrections for multiple comparison**

In light of the multiple statistical tests, we applied a more stringent *p*-value, by dividing the initial *p*-threshold (0.05) by 10, resulting in a threshold of *p*<0.005. However, since this was an exploratory analysis, results reaching the *p*<0.05 threshold are also reported, though not referred to as significant in the discussion.

**SUPPLEMENAL RESULTS**

**Demographics and peripheral iron markers**

The groups did not differ on pulse rate (t_81_=-0.39,*p*=0.699), systolic (t_81_=0.76,*p*=0.451) and diastolic blood pressure (t_81_=0.56,*p*=0.578), indicating that CUD patients were not acutely intoxicated.

There were no further significant relationships between brain iron and peripheral levels of ferritin, transferrin saturation, and serum iron (all *p*>0.5).

We did not observe relationships between QSM in GPi and the duration of cocaine use (CUD+O: *r*=.28,*p*=0.156; CUD−O: *r*=.26,*p*=0.372). In the CUD+O subgroup, who had been using cocaine for an average of 17.0 (±5.4) years and heroin for an average of 13.5 (±7.4) years, we observed a significant correlation between GPe and duration of cocaine use (*r*=.4.6,*p*=0.017) and a trend for a relationship with the duration of opiate use (*r*=.37,*p*=0.059). For the GPi there was no relationship with the duration of cocaine of opiate use (*r*=.32,*p*=0.108) in CUD+O.

There were no group differences with respect to GP (t_82_=0.67,*p*=0.505) or caudate volume (t_82_=-0.02,*p*=0.295), and no significant volume–QSM relationships in the GP (controls: *r*=0.13,*p*=0.425; CUD: *r*=-0.06,*p*=0.697) or the caudate (controls: *r*=-0.19,*p*=0.234; CUD: *r*=-0.18,*p*=0.262).

**SUPPLEMENAL TABLES**

**Table S1**: The QSM values calculated for each group across nine ROIs of iron-rich brain structures, which were manually traced on the QSM group template. For each ROI, the median QSM value was calculated. The tabulated values are the mean of these values for the control and cocaine groups.

|  | **Control Group** | | **Cocaine Group** | | **Group Comparison** | | |
| --- | --- | --- | --- | --- | --- | --- | --- |
|  | Mean | (±Std.) | Mean | (±Std.) | *t* | df | *p* |
| Caudate | 0.034 | (±0.007) | 0.035 | (±0.008) | -0.76 | 82 | 0.452 |
| Putamen | 0.043 | (±0.015) | 0.038 | (±0.012) | 1.65 | 82 | 0.103 |
| Pallidum external | 0.081 | (±0.011) | 0.092 | (±0.014) | -4.05 | 82 | <0.001 |
| Pallidum internal | 0.075 | (±0.012) | 0.083 | (±0.014) | -2.88 | 82 | 0.005 |
| Red nucleus | 0.066 | (±0.019) | 0.057 | (±0.018) | 2.14 | 82 | 0.036 |
| Substantia inominata | 0.084 | (±0.026) | 0.084 | (±0.022) | 0.18 | 82 | 0.858 |
| Substantia nigra | 0.072 | (±0.012) | 0.077 | (±0.012) | -1.71 | 82 | 0.091 |
| Dentate nucleus | 0.058 | (±0.021) | 0.059 | (±0.020) | -0.06 | 82 | 0.953 |
| Motor cortex | 0.020 | (±0.005) | 0.020 | (±0.005) | 0.25 | 82 | 0.803 |

**Table S2:** Group comparisons with respect to brain iron concentration (as indexed by QSM) and grey matter probabilities in both the putamen and GP respectively. For this direct comparison, we applied an automated, reproducible algorithm for subcortical segmentation to the MPRAGE template using FSL FIRST.

|  |  | **Putamen** | | | | **Globus Pallidus** | | | |
| --- | --- | --- | --- | --- | --- | --- | --- | --- | --- |
|  | **Group** | Mean (±Std.) | ***t*** | **df** | ***p*** | Mean (±Std.) | ***t*** | **df** | ***p*** |
| **QSM** | Control | 0.019 (±0.010) | 1.21 | 82 | 0.229 | 0.071 (±0.010) | -3.61 | 82 | 0.001 |
|  | Cocaine | 0.017 (±0.008) |  |  |  | 0.079 (±0.012) |  |  |  |
| **Volume** | Control | 0.325 (±0.070) | -2.56 | 82 | 0.012 | 0.008 (±0.006) | 0.67 | 82 | 0.505 |
|  | Cocaine | 0.367 (±0.079) |  |  |  | 0.007 (±0.008) |  |  |  |

**Table S3**: Summary of the findings in CUD patients in the putamen and GP. [*Age effects in the putamen refer to the lack of the normal age-related volume decline, age-related iron increase, and the relationship between the two; ** Familiarity effects are speculative, referring to published data showing abnormal volume increase in the putamen not only in CUD patients but also in their unaffected first-degree relatives].

|  | **Putamen** | **Globus Pallidus** |
| --- | --- | --- |
| **Grey matter volume** | Increased | normal |
| **Iron concentration** | Normal | increased |
| **Age effects*** | abnormal | N/A |
| **Drug effects** | none | positive relationship |
| **Familiarity effects**** | possible | unknown |

**Table S4:** Summary of regression analyses for variables predicting iron concentration in GPe (upper part of the table). Summary of hierarchical regression analyses for variables predicting iron concentration in GPi (lower part of the table). (R: Pearson product- moment correlation coefficients; B: β-coefficients; SEB: standard error of the β coefficients; β: standardized β coefficients for the regression model; R2: coefficients of determination; Δ: change; *p* < 0.05*; *p* < 0.001**).

| **QSM Globus Pallidus external**  Predicting variables | Unstandardized Coefficients | | Standardized Coefficients | *t* or *F* | *p* |
| --- | --- | --- | --- | --- | --- |
|  | B | SEB | ß |  |  |
| (Constant) | .078 | .004 |  | 17.64 | <0.001 |
| Cocaine dependence | .016 | .007 | .567* | 2.13 | 0.037 |
| Opiate dependence | -.014 | .004 | -.468** | -3.31 | 0.001 |
| Cannabis dependence | .006 | .004 | .169 | 1.48 | 0.143 |
| Alcohol dependence | -.012 | .007 | -.160 | -1.57 | 0.121 |
| Tobacco smoking status | .007 | .006 | .251 | 1.08 | 0.284 |
| Transferrin saturation | .000 | .000 | .149 | 1.42 | 0.159 |
| Serum ferritin levels | -4.019E-05 | .000 | -.187 | -1.71 | 0.092 |
| ΔR2 |  |  | .092** | 5.21 | <0.001 |
| **QSM Globus Pallidus internal**  Predicting variables | Unstandardized Coefficients | | Standardized Coefficients | *t* or *F* | *p* |
|  | B | SEB | ß |  |  |
| (Constant) | .073 | .005 |  | 15.028 | <0.001 |
| Cocaine dependence | .011 | .008 | .412 | 1.394 | 0.168 |
| Opiate dependence | -.011 | .005 | -.379* | -2.418 | 0.018 |
| Cannabis dependence | .004 | .004 | .112 | .887 | 0.378 |
| Alcohol dependence | -.009 | .008 | -.119 | -1.049 | 0.297 |
| Tobacco smoking status | .005 | .007 | .194 | .748 | 0.457 |
| Transferrin saturation | .000 | .000 | .078 | .670 | 0.505 |
| Serum ferritin levels | -1.308E-05 | .000 | -.061 | -.503 | 0.616 |
| ΔR2 |  |  | .175* | 2.245 | 0.040 |

**SUPPLEMENAL FIGURES**


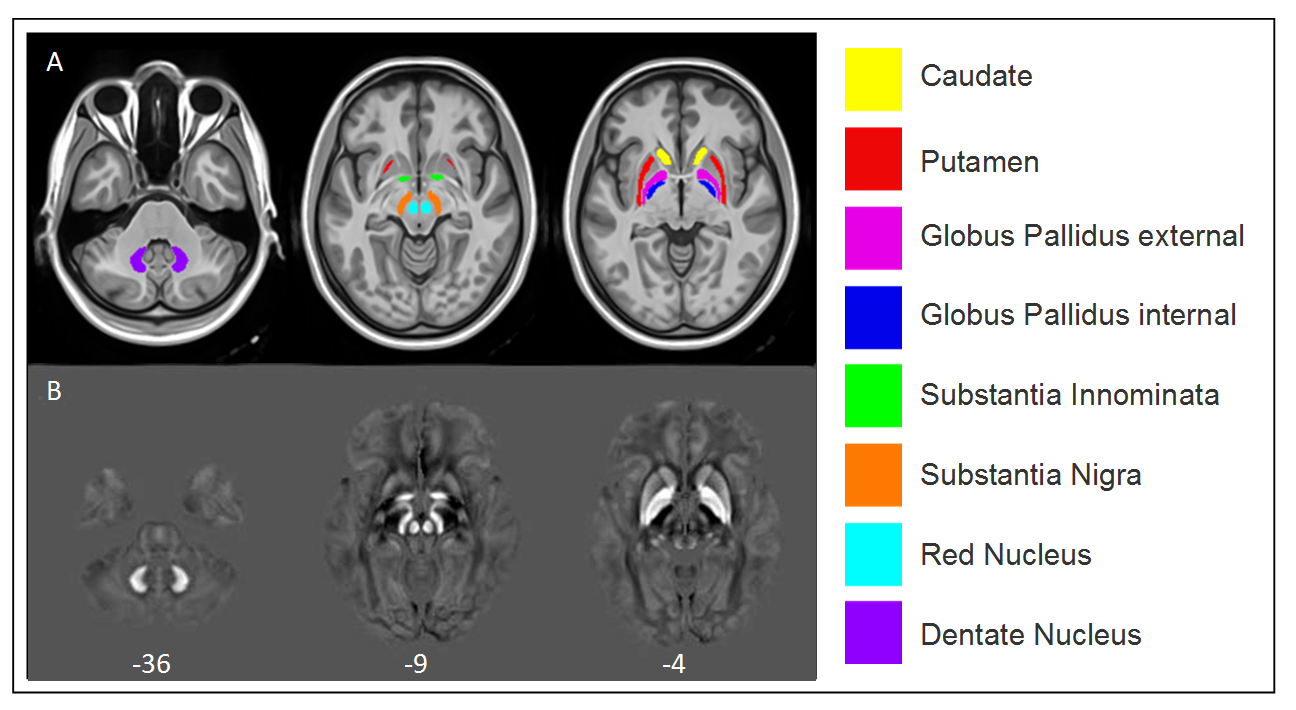


**Figure S1: (A)** Regions of interest (ROIs) of iron-rich subcortical brain structures, overlaid on an MPRAGE image. **(B)** The equivalent slices showing QSM data generated from a SWI dataset. High image intensity represents higher susceptibility (an index of iron concentration).


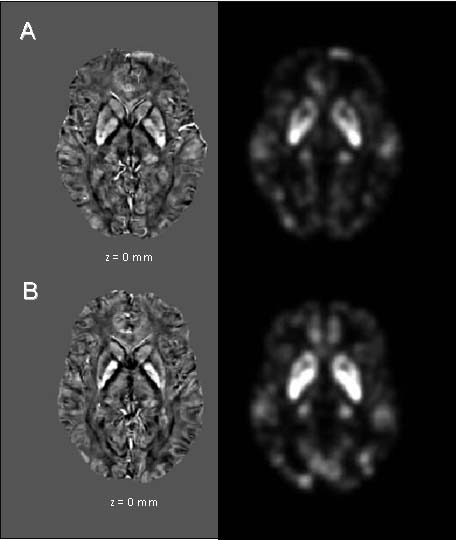


**Figure S2:** Example single-subject QSM images in MNI space (left side). QSM of the same individual following positive thresholding and smoothing (right side) **(A)** Control participant example. **(B)** CUD patient example. [Display range of left panels is -0.05 to 0.10, and right 0 to 0.05]

**+**
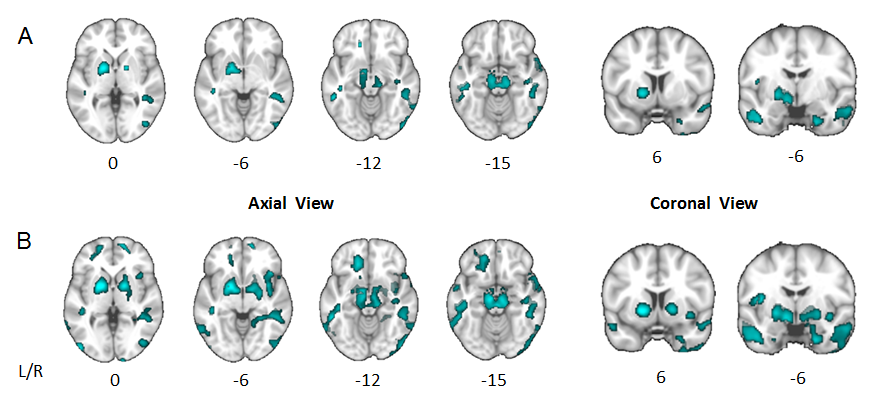


**Figure S3:** In order to determine whether *age* could explain some of the variance in our voxel-wise QSM data, we repeated the group comparison including *age* as a regressor. **(A)** The results of the original analysis, and **(B)** the results from the same data with *age* as a regressor in the design. As can be seen the spatial patterns of correlation are similar, if more extensive in the latter case. We have not used this approach in the main text however due to concerns that abnormal ageing effects in CUD might influence the results.

**SUPPLEMENAL REFERENCES**

1. Sheehan DV, Lecrubier Y, Sheehan KH et al. The Mini-International Neuropsychiatric Interview (MINI): The development and validation of a structured diagnostic psychiatric interview for DSM-IV and ICD-10. *J Clin Psychiat* 1998;**59**:22-33.

2. First MB, Spitzer RL, Gibbon M, Williams JBW. *Structured Clinical Interview for DSM-IV-TR Axis I Disorders, Research Version, Non-patient Edition. (SCID-I/NP)*. New York: Biometrics Research, New York State Psychiatric Institute, 2002.

3. Nelson HE. *National Adult Reading Test Manual*. Windsor (UK): NFER-Nelson, 1982.

4. Bohn MJ, Babor TF, Kranzler HR. The Alcohol-Use Disorders Identification Test (Audit) - Validation of A Screening Instrument for Use in Medical Settings. *J Stud Alcohol* 1995;**56**:423-432.

5. Skinner HA. The drug abuse screening test. *Addict Behav* 1982;**7**:363-371.

6. Mulligan AA, Luben RN, Bhaniani A et al. A new tool for converting food frequency questionnaire data into nutrient and food group values: FETA research methods and availability. *BMJ Open* 2014;**4**.

7. Hallberg L, Hulthen L. Prediction of dietary iron absorption: an algorithm for calculating absorption and bioavailability of dietary iron. *Am J Clin Nutr* 2000;**71**:1147-1160.

8. Kroot JJC, Laarakkers CMM, Geurts-Moespot AJ et al. Immunochemical and Mass-Spectrometry-Based Serum Hepcidin Assays for Iron Metabolism Disorders. *Clinical Chemistry* 2010;**56**:1570-1579.

9. Ersche KD, Barnes A, Jones PS, Morein-Zamir S, Robbins TW, Bullmore ET. Abnormal structure of frontostriatal brain systems is associated with aspects of impulsivity and compulsivity in cocaine dependence. *Brain* 2011;**134**:2013-2024.

10. Ersche KD, Jones PS, Williams GB, Turton AJ, Robbins TW, Bullmore ET. Abnormal Brain Structure Implicated in Stimulant Drug Addiction. *Science* 2012;**335**:601-604.

11. Acosta-Cabronero J, Betts M, Cardenas-Blanco A, Yang S, Nestor PJ. In vivo MRI mapping of brain iron deposition across the adult lifespan. *J Neurosci* 2016;**36**:364-374.

12. Daugherty A, Raz N. Age-related differences in iron content of subcortical nuclei observed in vivo: A meta-analysis. *Neuroimage* 2013;**70**:113-121.

13. Berlow YA. MRI assessment of brain iron content in methamphetamine users. 2014.; http://digitalcommons.ohsu.edu/etd/3541/ assessed on 10 July 2016

14. Smith SM. Fast robust automated brain extraction. *Hum Brain Mapp* 2002;**17**:143-155.

15. Walsh DO, Gmitro AF, Marcellin MW. Adaptive reconstruction of phased array MR imagery. *Magn Reson Med* 2000;**43**:682-690.

16. Schofield MA, Zhu YM. Fast phase unwrapping algorithm for interferometric applications. *Optics Letters* 2003;**28**:1194-1196.

17. Schweser F, Deistung A, Lehr BW, Reichenbach JR. Quantitative imaging of intrinsic magnetic tissue properties using MRI signal phase: An approach to in vivo brain iron metabolism? *Neuroimage* 2011;**54**:2789-2807.

18. Liu T, Wisnieff C, Lou M, Chen W, Spincemaille P, Wang Y. Nonlinear formulation of the magnetic field to source relationship for robust quantitative susceptibility mapping. *Magn Reson Med* 2013;**69**:467-476.

19. Avants BB, Epstein CL, Grossman M, Gee JC. Symmetric diffeomorphic image registration with cross-correlation: Evaluating automated labeling of elderly and neurodegenerative brain. *Medical Image Analysis* 2008;**12**:26-41.

20. Tustison NJ, Avants BB, Cook PA et al. N4ITK: Improved N3 Bias Correction. *Ieee Transactions on Medical Imaging* 2010;**29**:1310-1320.
